# *Seeing* and *looking*: Evidence for developmental and stimulus-dependent changes in infant scanning efficiency

**DOI:** 10.1371/journal.pone.0274113

**Published:** 2022-09-16

**Authors:** Shannon Ross-Sheehy, Bret Eschman, Esther E. Reynolds

**Affiliations:** 1 Department of Psychology, University of Tennessee, Knoxville, TN, United States of America; 2 Department of Psychology, University of Tennessee at Chattanooga, Chattanooga, TN, United States of America; University of Iowa, UNITED STATES

## Abstract

Though previous work has examined infant attention across a variety of tasks, less is known about the individual saccades and fixations that make up each bout of attention, and how individual differences in saccade and fixation patterns (i.e., scanning efficiency) change with development, scene content and perceptual load. To address this, infants between the ages of 5 and 11 months were assessed longitudinally (Experiment 1) and cross-sectionally (Experiment 2). Scanning efficiency (fixation duration, saccade rate, saccade amplitude, and saccade velocity) was assessed while infants viewed six quasi-naturalistic scenes that varied in content (social or non-social) and scene complexity (3, 6 or 9 people/objects). Results from Experiment 1 revealed moderate to strong stability of individual differences in saccade rate, mean fixation duration, and saccade amplitude, and both experiments revealed 5-month-old infants to make larger, faster, and more frequent saccades than older infants. Scanning efficiency was assessed as the relation between fixation duration and saccade amplitude, and results revealed 11-month-olds to have high scanning efficiency across all scenes. However, scanning efficiency also varied with scene content, such that all infants showing higher scanning efficiency when viewing social scenes, and more complex scenes. These results suggest both developmental and stimulus-dependent changes in scanning efficiency, and further highlight the use of saccade and fixation metrics as a sensitive indicator of cognitive processing.

## Introduction

Rudimentary visual scanning is apparent from the first moments of life [1]. Over the first several postnatal weeks, the frequency and speed of eye movements, or *saccades*, increase [2, 3] along with rapid improvements in acuity [4], contrast sensitivity [5], and visual attention [6–8]. Without a doubt, the timing of these maturational improvements is crucial, and this development interacts with experience in important ways. For example, early visual experience as a result of preterm birth may result in relatively fast shift rates [9] and altered patterns of spatial attention [10], both of which may have lasting effects on behavior and attentional functioning [11].

As vision continues to improve over the first months of life, eye movements become more volitional, with increased fixations to both *semantically* salient features such as the eyes or mouth [12–14], and *perceptually* salient features such as high contrast object boundaries [15, 16]. The development of attention also influences saccade and fixation dynamics, and previous research suggests that typical development proceeds from slow visual orienting and sparse visual scanning, to fast, efficient visual orienting with increased visual scanning [8, 17–19]. This shift is likely driven by neural maturation [20], increased volitional or “top down” control of eye movements [6, 21] and improvements in rapidly disengaging attention and re-fixating eyes in a new location [19]. Although many factors influence the kind of things infants look at, the speed of the eye movement system approaches adult levels by around 7 months [2] at around 1.7 to 3 saccades per second [22].

Although the development of the eye movement system is relatively well-understood, most previous work has focused on gaze duration as a measure of cognitive development. For example, most tasks find mean or median gaze or *look* durations generally decrease with development [23–28], and these changes are thought to reflect improvements in memory, attention and processing speed. In addition to these general developmental patterns, measures based on look duration demonstrate clear individual differences [25, 29]. For example, 4-month-old infants who showed relatively short *peak* look durations in the context of an infant-controlled familiarization task (i.e., “short lookers”) produced qualitatively different patterns of learning than “long lookers” [25, 29]. Thus, look duration appears to be a relatively sensitive indicator of individual differences in things like encoding and/or processing speed [29, 30], and these differences appear to be relatively stable [23].

It is important to note that look duration is often measured in the context of a familiarization or habituation task, in which an experimenter watches the baby as they engage with the stimulus, coding a *look* when infants orient gaze toward the display for at least one second, and coding a *look away* when the infant turns away from the display for more than one second [31, 32]. Thus, overall visual engagement is typically parsed into bouts of “holistic attention” (oriented gaze toward the stimulus) punctuated by disengagement (looks away from the stimulus), and look duration is calculated based on these individual bouts of holistic attention. This approach is particularly well-suited to video or live coding, where individual saccades and fixations cannot be easily discerned. Although measures based on holistic attention provide an important index of cognitive processing, each look is comprised of numerous individual fixations and saccades. Thus, it is unclear if previous developmental findings were driven by changes in holistic attention, or by underlying changes in the saccades and fixations that make up bouts of holistic attention. To address this question, it will be necessary to utilize eye tracking to visualize individual fixations and saccades, as it has higher spatial and temporal resolution compared to using human observers [33, 34].

### Holistic attention and scanning dynamics

Much is known about the kinds of things that elicit attention [17, 35, 36], as well as the relation between holistic attention and cognition [25, 29]. However relatively little is known about the patterns of saccades and fixations that make up each bout of holistic attention, and how this is related to efficient information processing. Work with adults may shed some light on this. For example, in one visual search task [37] participants were divided up into two groups. One group was instructed to *actively* search the display to find the target, whereas the other group was instructed to *passively* view the display, allowing the target to “pop into mind”. Authors reasoned that the active strategy required executive control of gaze and attention, whereas the passive condition allowed participants to rely on more implicit or automatic processing, processes which likely dominate young infant scanning behavior. Both groups were equally accurate in finding the target. However, adults in the passive condition were faster to find the target and produced fewer fixations that were longer in duration with larger subsequent saccades than the active searchers [37]. Authors suggested these findings highlighted the difference between “looking” and “seeing”, demonstrating the critical role of automatic processes in fixation and saccade dynamics. These findings also underline the importance of both fixation and saccades and their role in visual cognition; a relationship that was well-characterized here:

“Fixations during visual search cannot be considered in isolation; they are always involved in a trading relationship with saccades. That is, at any given moment the observer is engaged in strategic decisions (albeit implicit ones) to keep their eyes still (allowing for seeing, the ability to distinguish targets from non-targets) or to move them (allowing for looking, the acquisition of new information from outside of the current fixation).” [38]

Watson and colleagues [37] hypothesized that passive looking or “seeing” strategies prioritize local processing, with longer fixation durations leading to more complete encoding of proximal area, enabling larger saccades away from the current locus of attention. Although both “looking” and “seeing” search strategies were observed in the context of an *explicit* search task (i.e., the participants were tasked with finding the target), the passive “seeing” strategy relies heavily on automatic processing. Thus, it may be possible to observe this scanning strategy even without an explicit task, such as when infants passively view a novel scene. In addition to these task-dependent changes in the saccade/fixation relation, there are also robust individual differences in saccade properties. For example, participants who are particularly good at visual foraging tasks tend to produce smaller saccades overall, and are less likely to revisit previous locations relative to unskilled foragers [39].

Thus, previous research suggests that saccade and fixation dynamics change as a function of volitional or automatic attention processes [38] with smaller and less frequent saccades related to increased search efficiency [39]. Given saccade and fixation metrics are likely to change with development, it is possible that individual differences observed using holistic attention tasks may be driven to some extent by underlying changes in scanning efficiency. For example, based on the slow-to-fast developmental progression typically observed in the context of familiarization tasks [25], we might expect relatively long fixation durations for younger infants compared to older infants. In addition, we might expect saccade amplitudes to be slightly shorter for younger infants, based on previous findings of hypometric saccades in young infants [40]. We also might expect saccades to be less frequent for younger infants, based on findings that younger infants orient more slowly when multiple objects compete for attention [19, 41].

We present here results from a passive viewing task in which infants briefly viewed six quasi-naturalistic scenes, that is, scenes that *looked* realistic (e.g., people or objects in a room) but that controlled for particular properties, such as the number of subjects/objects per scene. Experiment 1 was designed as an *assessment task*, thus all subjects received the exact same stimulus order. This ensured performance was comparable across individuals and across three longitudinal test sessions at 5, 8 and 11 months. Experiment 2 was designed as an *experimental task* with a cross-sectional design, random trial presentation, longer trial durations, and increased control of stimulus properties such as eccentricity, density, and focal region. Replicating these tasks both longitudinally and cross-sectionally enabled us to focus only those scanning metrics that were *consistent* across age and task variations, and that produced *robust* individual differences. In addition, using different commercial eye trackers for each experiment (Tobii TX-300 and Eyelink 1000+) increased confidence that results were not driven by low-level differences in sample rate, calibration protocols, and/or saccade and fixation parsers.

## Experiment 1

**Fig 1:**
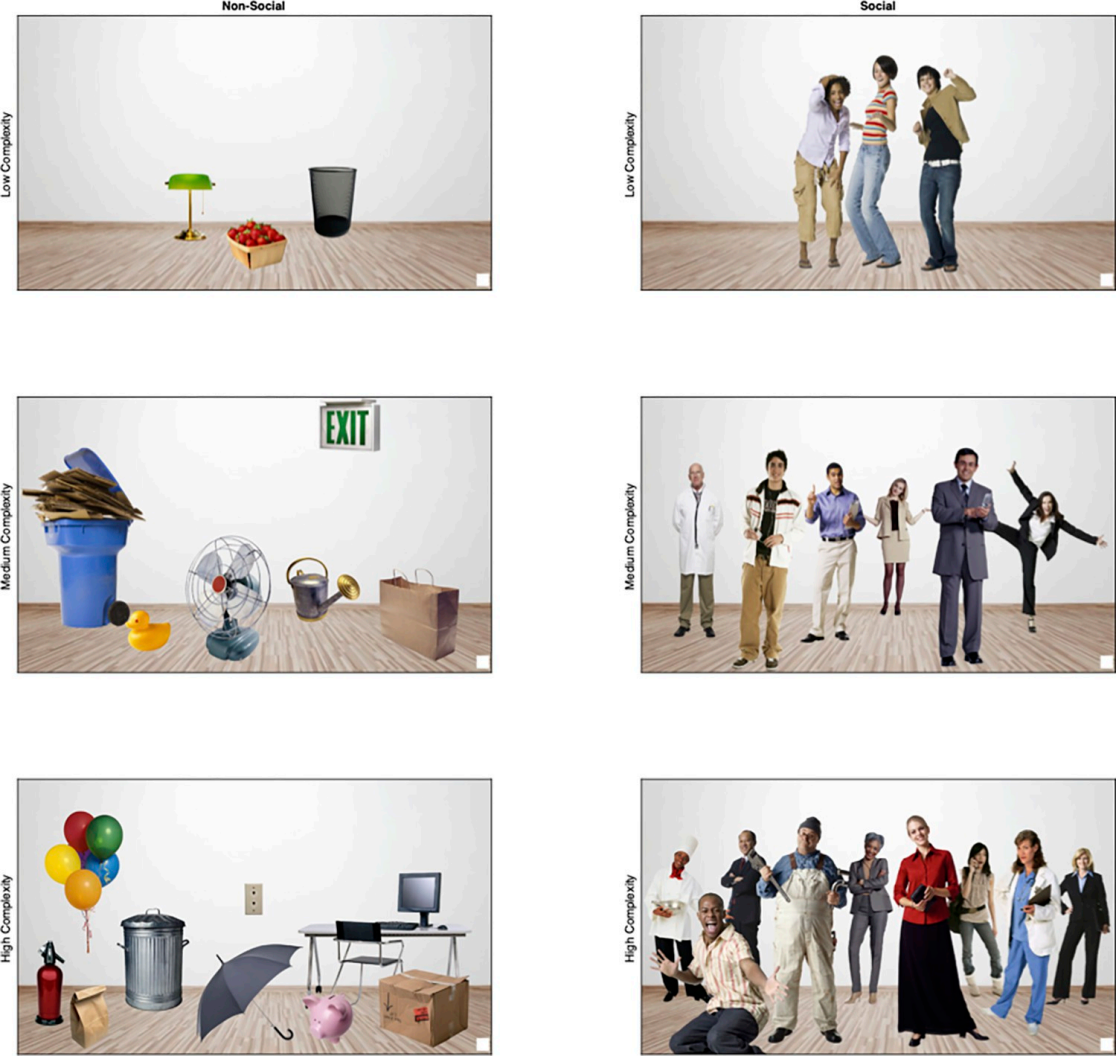
Quasi-naturalistic stimuli used in Experiment 1. Scenes varied by content: *non-social* (left column) or *social* (right column) and complexity: *low* (top row), *medium* (middle row) and *high* (bottom row).

The primary aim of Experiment 1 was to determine if individual differences in saccade and fixation dynamics are stable over time. To accomplish this, we tested infants longitudinally at 5, 8, and 11 months, and conducted growth curve analyses to assess the stability of the average number of saccades per second (*saccade rate*), the average duration of gaze between eye movements (*fixation duration*) and the average length of each eye movement (*saccade amplitude*). The secondary aim was to determine if saccade patterns varied across broad classes of stimuli, and if this relation changed over development. To accomplish this, we created quasi-naturalistic scenes in a fully crossed design that included a scene content manipulation (social or non-social) as well as scene complexity manipulation (low, medium or high, see Fig 1). Our focus in creating these stimuli was to maximize power for our growth curve analysis by using everyday visual stimuli that were interesting enough to produce multiple saccades and fixations, and robust enough to be used across a wide range of ages. Stimuli were created to be maximally effective in eliciting saccades, and all contained high contrast luminance contours where the focal point (objects or people) met the white background. This is in contrast to typical naturalistic stimuli, in which the foreground and background are differentiated by relatively subtle chromatic and luminance contrasts (e.g., photographs of landscapes or cluttered rooms). Note: Social scenes for both Experiments 1 and 2 were created using Microsoft Office 2011 Clipart, and their non-commercial use is covered under the Microsoft End User License Agreement (EULA).

As is typical for assessment tasks, we incorporated a fixed-order design to ensure that performance could be directly compared across individuals and visits. Infants were first presented with non-social stimuli (low to high), then social stimuli (low to high), because previous behavioral reactivity work suggested a great deal of variability in infants’ tolerance for highly complex or highly social stimuli [42, 43]. We kept the trial durations very short to ensure infants would view every scene for each of their three visits. Though others have used trials as short as 5 seconds for similar naturalistic scenes [44], pilot testing revealed 7 second trials ensured sufficient scanning time, while preventing familiarization effects that might lead some infants to disengage from the stimuli.

### Methods

#### Participants

Infants were tested longitudinally at 5, 8 and 11 months. These ages were selected based on previous work suggesting this range would capture key developments in visual attention and scanning [6, 29, 45]. Infant names were obtained from the Tennessee Department of Health, and all infants were full term (gestational age > = 37 weeks) with no reported birth defects or vision problems. Participants included 157 infants who completed a total of 292 test sessions. Of these 292 sessions, 12 were excluded due to fussiness, 3 due to sleepiness, 12 due to inability to calibrate, 5 due to missing data for two or more conditions, and 10 due to equipment failure. These eliminations left an additional 65 infants with only a single valid test session, all of whom were subsequently removed. This resulted in a final sample of 74 infants who had completed at least two of the three test sessions, for a total of 175 sessions. Of the 74 infants, 28 participated at all three ages, 2 participated at 5 and 8 months, 23 participated at 5 and 11 months, and 21 participated at 8 and 11 months. All infants were within ± 11 days of the target age. The age distributions were as follows: 5 months (*n* = 53, *M* = 154.28 days, *SD* = 8.61, 29 male, 24 female), 8 months (*n* = 50, *M =* 246.18 days, *SD* = 7.57, 30 male, 20 female), and 11 months (*n* = 72, *M* = 333.96 days, *SD* = 8.61, 42 male, 30 female). Parent reported race and ethnicity for the final sample indicated that 60 of the infants were White (3 Hispanic, 55 Non-Hispanic, 2 declined to answer), 9 were Multi-Racial, 1 was Black, 1 was Middle Eastern, and 2 declined to answer. Nine of the mothers reported having a high school degree or equivalent, 22 of the mothers reporting having some college or a 2-year degree, 27 had a 4-year degree, 13 had a master’s or professional degree, and 3 had a doctoral degree. Parents received a $20 gift card and infants received a small toy.

#### Stimuli and procedure

The following procedures were approved by the East Tennessee State University Institutional Review Board (IRB-0314.29s). Written informed consent was obtained from the parent/guardian prior to the testing session. Stimuli were presented on a 23” 60 Hz monitor with a viewable surface of 40.2° (w) by 30.9° (h) at a distance of 65 cm. Participants were tested in a dimly lit room and eye movements were recorded as they viewed each of six trials, three social, three non-social, at varying levels of complexity (low = 3 objects or people, medium = 6 objects or people, high = 9 objects or people, Fig 1), complexity defined as the number of objects or people in the scene. Trials were presented in a fixed order to reduce likelihood of fussing due to individual differences in tolerance of social or complex scenes, and to ensure that all infants saw the exact same events for each subsequent visit [42, 46]. Trials progressed from least to most interesting, starting first with non-social scenes (low, medium, high), followed by social scenes (low, medium, high) and lasted 7 seconds each. Each trial began with a dynamic audiovisual central fixation stimulus, and the trial was started when an experimenter seated out of sight judged the infant to be looking.

#### Eye tracking and data reduction

Continuous gaze was collected throughout the session using a Tobii TX300 eye tracker. Infants were binocularly calibrated using the 5-point infant calibration scheme with dynamic calibration stimuli. Raw gaze coordinates were sampled at 300 Hz using the infant illumination mode, and fixations and saccades were parsed using the Tobii Studio I-VT velocity filter with settings designed to adapt the default adult parameters to appropriate infant values [47, 48]: Gaze was interpolated using a 75ms max gap, and the signal from both eyes was averaged. Samples in which only a single eye was detected were discarded. Data were smoothed using a 7-sample moving median which is more resistant to outliers. Velocity was calculated over 40ms window length, and movement exceeding 40 degrees/second was classified as a saccade. Adjacent fixations were merged (max gap 75ms, max angle 1 degree), and looks shorter than 60ms were discarded as noise. Saccade rate (the mean number of saccades per second) and mean fixation duration in ms were calculated using a custom MATLAB script. Sixteen participants were missing gaze data from a single trial due to eye tracker noise, and these missing cells were filled with the series mean for each age. This preserved the age and condition means for each dependent measure, while allowing us to retain the participants, substantially increasing power for our growth curve analysis. Means, standard deviations, and sample sizes for all measures are presented in Table 1.

**Table 1:** Means, standard deviations, and sample sizes for all measures in Experiment 1 and Experiment 2.

				Measures					
				Saccade Rate (s)		Mean Fixation Duration (ms)		Saccade Amplitude (deg)	
Experiment	Age	*n*	Content (Complexity)	*M*	*SD*	*M*	*SD*	*M*	*SD*
1	5 Months	53	Non-Social (Low)	2.64	1.59	399.58	205.43	5.22	1.52
			Non-Social (Med)	2.52	1.74	339.51	171.00	8.23	2.89
			Non-Social (High)	2.03	1.22	447.25	255.95	9.09	3.01
			Social (Low)	1.69	0.95	557.82	393.30	4.86	1.92
			Social (Med)	1.70	0.95	495.03	237.57	6.38	2.11
			Social (High)	1.49	0.81	494.12	287.59	6.38	2.93
	8 Months	50	Non-Social (Low)	1.95	1.22	500.11	314.96	5.36	1.79
			Non-Social (Med)	2.01	0.98	565.25	672.66	8.53	2.39
			Non-Social (High)	1.77	0.67	425.94	148.45	8.10	2.68
			Social (Low)	1.38	0.51	532.83	196.63	4.98	2.16
			Social (Med)	1.36	0.52	588.52	282.66	6.37	2.34
			Social (High)	1.44	0.67	529.91	177.83	6.15	1.93
	11 Months	72	Non-Social (Low)	1.74	0.86	447.83	185.71	5.33	1.27
			Non-Social (Med)	1.85	0.85	448.99	446.87	7.36	2.68
			Non-Social (High)	1.52	0.68	463.50	315.46	8.33	2.42
			Social (Low)	1.39	0.61	541.85	207.80	4.81	1.44
			Social (Med)	1.41	0.58	510.69	169.74	6.58	2.02
			Social (High)	1.32	0.62	565.34	243.95	6.63	1.86
2	5 Months	24	Non-Social (Low)	2.06	0.53	412.26	141.61	8.15	2.66
	* *		Non-Social (Med)	2.09	0.58	377.30	131.20	8.73	3.25
			Non-Social (High)	2.34	0.75	346.06	124.96	9.66	4.05
			Social (Low)	1.84	0.73	451.29	179.78	6.83	2.14
			Social (Med)	1.91	0.58	415.15	121.84	8.29	3.35
			Social (High)	1.93	0.38	430.34	103.44	7.98	2.58
	7 Months	24	Non-Social (Low)	1.81	0.89	437.09	181.19	6.79	3.08
	* *		Non-Social (Med)	1.82	0.84	382.68	99.87	9.07	2.41
			Non-Social (High)	2.09	0.57	360.77	67.93	7.73	2.42
			Social (Low)	1.56	0.53	494.47	191.68	5.87	2.23
			Social (Med)	1.80	0.75	447.75	157.41	7.47	3.22
			Social (High)	1.67	0.69	568.90	394.43	7.02	2.88
	11 Months	24	Non-Social (Low)	1.74	0.47	390.17	91.13	6.13	2.02
	* *		Non-Social (Med)	2.09	0.38	357.18	58.20	7.34	1.88
			Non-Social (High)	2.03	0.53	376.24	94.88	7.17	2.01
			Social (Low)	1.53	0.56	501.69	154.25	5.74	2.04
			Social (Med)	1.65	0.35	464.52	110.09	6.20	2.01
			Social (High)	1.57	0.48	534.13	200.01	5.46	1.76

### Results

#### Linear mixed effects models

The primary aims of Experiment 1 were to determine if individual differences in saccade and fixation dynamics were stable from 5- to 11-months-of-age, and to determine if scanning patterns vary across broad classes of stimuli. To accomplish these aims, linear mixed effects (LME) models were used to fit unconditional and conditional growth curves to our longitudinal data [49; R package lme4]. LME models have several important benefits over repeated measures ANOVAs for modeling longitudinal and repeated measures data, including their ability to produce robust estimates despite missing session data, and their ability to capture both fixed effects (i.e., conditional means) and random effects (individual deviations from the conditional means). This allowed us to embed subject-specific changes over time (i.e., slope) within the larger overall regression model [50].

We created three candidate models for each measure and selected the simplest model that captured the greatest amount of variability. Our first model served as our baseline model (m0), and included a fixed effect of age, and a random effect of age within subject (i.e., random subject-level slopes and intercepts). Our next model (m1) started with the baseline model and added additional fixed effects for content and complexity. Our final model (m2) started with model 1, and added interaction effects for age, content, and complexity (Table 2). Age and complexity were coded as continuous variables, and content was categorical (content was deviation coded so the constant represents the grand mean, and coefficients can be interpreted as main effects). If development is the most important factor in driving change on a visual scanning task, then we would expect our baseline model (m0) to produce the best model fits. If, however, stimulus content (i.e., learning/memory) and complexity (i.e., visual competition) are also important, then we would expect to see improved model fits for our second model (m1). Finally, if the effects of content and complexity vary with age, or if scene content and complexity themselves interact, we expect our last model to produce the best fits (m2).

**Table 2:** Experiment 1 estimates and standard error for all model effects. Significant effects for best-fitting model noted in bold.

	Model Comparisons									
	Saccade Rate (s)				Mean Fixation Duration (ms)			Saccade Amplitude (deg)		
Constant		2.16***	2.35***	**2.61** ***	465.75***	**471.51** ***	504.76***	6.80***	4.44***	**4.19** ***
		(0.17)	(0.18)	**(0.23)**	(32.47)	**(38.58)**	(64.71)	(0.23)	(0.27)	**(0.49)**
Age		-0.22***	-0.22***	**-0.34** ***	13.71	13.54	-2.33	-0.10	-0.10	0.02
		(0.06)	(0.06)	**(0.09)**	(11.92)	(11.99)	(27.35)	(0.09)	(0.09)	(0.21)
Content			0.52***	**1.24** ***		**-86.97** ***	-128.72*		1.34***	0.38
			(0.05)	**(0.17)**		**(16.83)**	(61.41)		(0.13)	(0.48)
Complexity			-0.10***	**-0.23** ^**^		-2.69	-19.31		1.18***	**1.31** ***
			(0.03)	**(0.08)**		(10.31)	(27.88)		(0.08)	**(0.22)**
Age*Content				**-0.22** ***			14.98			**-0.33** *
				**(0.06)**			(20.07)			**(0.16)**
Age*Complexity				0.06			7.88			-0.06
				(0.03)			(12.29)			(0.10)
Content*Complexity				**-0.13** ^*^			5.08			**0.82** ***
				**(0.06)**			(20.60)			**(0.16)**
Model	m0		m1	**m2**	m0	**m1**	m2	m0	m1	**m2**
ChiSq Test	--		*p* < .001	***p* < .001**	--	***p* < .001**	*p* = .794	--	*p* < .001	***p* < .001**
Best Fit	no		no	**yes**	no	**yes**	no	no	no	**yes**
Observations	1,050		1,050	**1,050**	1,050	**1,050**	1,050	1,050	1,050	**1,050**
Log Likelihood	-1,372.79		-1,312.72	**-1,301.26**	-7,446.89	**-7,433.370**	-7,433.19	-2,476.06	-2,344.56	**-2,329.58**
Akaike Inf. Crit.	2,757.57		2,641.45	**2,624.52**	14,905.78	**14,883.40**	14,888.37	4,964.11	4,705.11	**4,681.15**
Bayesian Inf. Crit.	2,787.31		2,681.10	**2,679.04**	14,935.52	**14,923.05**	14,942.89	4,993.85	4,744.76	**4,735.68**

Note:

*p<0.05

**p<0.01

***p<0.001

Chi-square goodness of fit tests (***χ***^**2**^) were conducted comparing the current model (m_*n*_) to the previous model (m_*n-1*_). Results of this analysis are reported in Table 2, along with three additional model fit metrics: Log likelihood, Akaike information criterion (AIC), and Bayesian Information Criterion (BIC). Both AIC and BIC incorporate a penalty on model complexity to help prevent “overfitting”. Lower values for AIC and BIC indicate better model fits, higher values for log likelihood indicate better model fits.

#### Saccade rate

**Fig 2:**
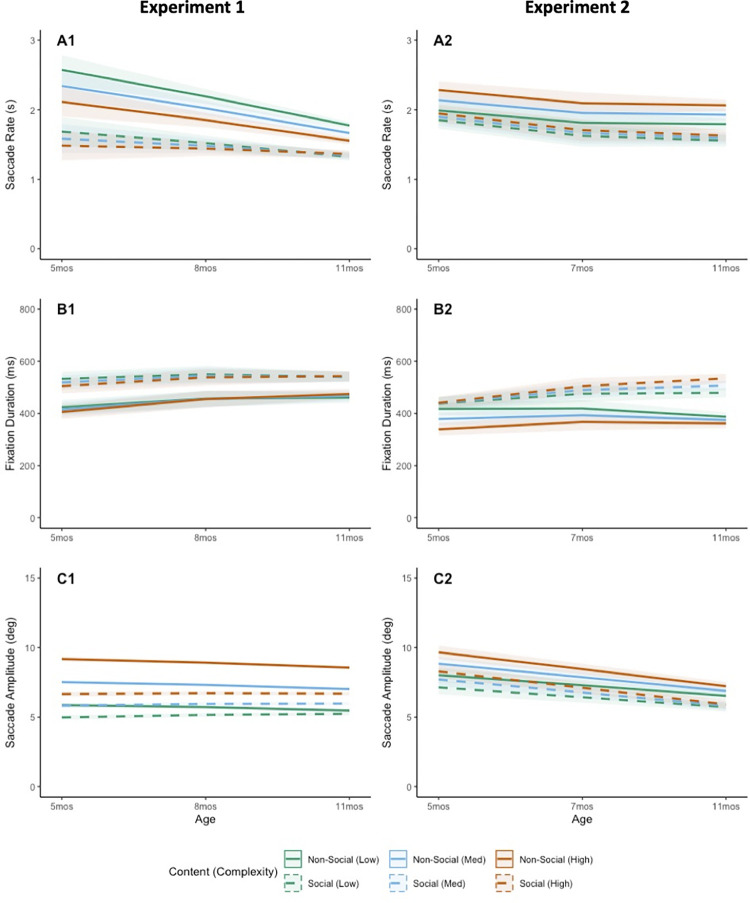
Fitted model estimates by condition for Experiment 1 (left) and Experiment 2 (right). Measures include average *saccade rate* (A), average *fixation duration* (B), and average *saccade amplitude* (C). Shading indicates 95% confidence intervals.

The number of saccades per second (saccade rate) was calculated for each subject and condition as the sum of all saccades divided by the trial duration. Results of our model fitting revealed that the majority of fit metrics converged on model 2 (Table 2). Chi-square analysis further confirmed that model 2 provided a significantly better fit than model 1 (*p* < .001), suggesting important interaction effects. To probe these differences further, we next examined the model estimates for the best-fitting model (m2). As can be seen in Table 2, results for saccade rate revealed a significant complexity main effect, with saccade rates decreasing as complexity increased (Fig 2, panel A). Results also revealed significant age and content main effects, qualified by a significant age by content interaction. Although all infant saccade rates decreased with age, this effect was particularly apparent for the non-social stimuli. Finally, results revealed a significant content by complexity interaction, with lowest saccade rates for high complexity social scenes.

#### Fixation duration

Mean fixation duration was calculated for each subject and condition as the average duration of each stable gaze event that was flanked on either side by saccades. Results of our model fitting revealed the majority of model fit metrics to converge on model 1 (Table 2), and our chi-square analysis confirmed that model 1 provided a significantly better fit than model 0 (*p* < .001), and model 2 was not significantly better than model 1 (*p* = .794). This suggests that age did not interact with either content or complexity, and that content and complexity did not themselves interact. Indeed, estimates derived from our best-fitting model (m1) reveal only a significant main effect of content, with significantly longer mean fixation durations when viewing social stimuli (Fig 2, panel B). Interestingly, there were no significant age effects, which is somewhat surprising given frequent findings of decreasing fixation duration with increasing age. Complexity similarly did not influence fixation durations.

#### Saccade amplitude

Mean saccade amplitudes were calculated for each subject and condition as the average length of each saccade in degrees. Results of our model fitting revealed that the majority of fit metrics converged on model 2 (Table 2). Chi-square analysis further confirmed that model 2 provided a significantly better fit than model 1 (*p* < .001), suggesting important interaction effects. An examination of model estimates for our best-fitting model (m2) revealed a significant complexity main effect which was subsumed under a significant content by complexity interaction, with largest saccade amplitudes for medium and high non-social scenes. We also see a significant age by content interaction, with average saccade amplitudes to non-social stimuli decreasing slightly relative to social stimuli (Fig 2, panel C). This suggests scanning strategies may shift with age; though overall saccade lengths are larger for non-social stimuli at every age, the difference between the two categories becomes less apparent with age.

#### Stability over time

Before examining the results, it is important to clarify how stability is conceptualized in this research project. Stability over time can be thought of as infant’s internal reliability; in other words, the degree to which each infant is showing consistent patterns of performance in relation to their previous scores and the scores of the other participants. To assess the stability of these metrics across development, we conducted a Pearson bivariate correlation analysis for each of our three scanning metrics, and results are presented in Table 3. Looking first to measures of stability, we see that saccade rates showed moderate stability from 5 to 11 months, *r*(50) = .307, *p* = .027, and mean fixation duration showed strong stability from 8 to 11 months, *r*(47) = .511, *p* < .001. Saccade amplitudes showed the stability, with moderate to high stability from 5 to 8 months, *r*(28) = .472, *p* = .008, and from 8 to 11 months, *r*(47) = .380, *p =* .007.

**Table 3:** Experiment 1 correlation table for all scanning metrics across 5-, 8-, and 11-month longitudinal visits. Sample sizes for comparison groups were as follows: 5 and 8 months (*n* = 30), 5 and 11 months (*n* = 52), and 8 and 11 months (*n* = 49). Significant correlations noted in bold.

		Correlations										
		Saccade Rate			Mean Fixation Duration				Saccade Amplitude			
		5m	8m	11m		5m	8m	11m		5m	8m	11m
Saccade Rate (s)	5m	--										
	8m	.223	--									
	11m	**.307** ^*^	0.031	--								
Mean Fixation Duration (ms)	5m	**-.608** ^**^	-0.138	0.060		--						
	8m	-.099	**-0.357** ^*^	-0.027		0.268	--					
	11m	-.200	-0.210	**-0.380** ^**^		0.247	**0.511** ^**^	--				
Saccade Amplitude (deg)	5m	-.242	0.131	-0.205		0.059	0.067	0.234		--		
	8m	**-.547** ^**^	-0.150	**-0.308** ^*^		0.297	0.033	0.114		**0.472** ^**^	--	
	11m	**-.332** ^*^	-0.023	-0.053		0.251	0.166	0.179		0.240	**0.380** ^**^	--

Note:

*p<0.05

**p<0.01

***p<0.001

Moreover, an examination of *within-age* relations between saccades and fixations reveals a strong negative correlation between saccade rate and fixation duration at every age: 5 months, *r*(52) = -.608, *p* < .001, 8 months, *r*(51) = -.357, *p* = .010, and 11 months, *r*(71) = -.380, *p* < .001. This is not surprising, and suggests that overall, infants who have more frequent eye movements also tend to make shorter fixations.

Finally, an examination of *between-age and measure* relations reveals strong negative correlations between 5-month-old saccade rate and saccade amplitude at both 8 months, *r*(28) = -.547, *p* = .002, and 11 months, *r*(50) = -.332, *p* = .016. This makes sense and suggests that infants with high numbers of saccades at 5 months tend to make the shortest saccades at 8 and 11 months. This also bolsters our confidence that our high saccade rates at 5 months were not driven by eye tracker noise, as we would not expect these relations to hold across age if that were the case.

### Discussion

Model results reveal several important effects. First, individual differences in saccade rates are relatively stable over time, and younger infants make significantly more saccades than older infants. This is somewhat surprising and suggests that young infants may struggle to maintain focus, particularly as the scenes increased in complexity. This is consistent with the significant negative correlation between saccade rates and fixation duration, with high scanning infants producing the shortest fixation durations. It is interesting that the bulk of these saccades are occurring for medium and high complexity non-social scenes, where objects are more diverse and spread throughout the stimulus space. It is possible that these scenes produced qualitatively different saccade patterns for our youngest infants due to novelty, eccentricity, or some other perceptual factor. This possibility will be explored in Experiment 2, using a more tightly controlled stimulus set.

Across all ages we saw strong evidence of content effects on scanning, with significantly lower saccade rates, significantly longer fixation durations, and significantly shorter saccade amplitudes for social scenes compared to non-social scenes. Although these effects are highly consistent, caution is warranted as some aspects of our naturalistic scenes could have contributed to these condition effects. For example, it is possible that content and complexity effects were influenced by low-level “salience” differences brought about by differences in clustering, total eccentricity, and/or spatial location for the social and non-social stimuli. For example, our finding of significantly longer saccade amplitudes for medium and high complexity non-social scenes, might have been influenced by differences in total eccentricity and feature density for non-social compared to social stimuli (Fig 2 panel C). It is also possible that content effects in both measures were an artifact of our fixed trial design, with interest waning as the task progressed. These possibilities will also be examined in Experiment 2.

Perhaps the most surprising finding is the lack of age effects in our mean fixation duration measure, although others have observed a similar null effect in the same age range [44]. This suggests either that mean fixation duration is qualitatively distinct from measures derived using holistic attention, or that our short trial durations were simply too brief to see the kinds of looking differences we might capture with a standard familiarization task. This possibility will be further explored in Experiment 2. This finding along with strong content and complexity effects for our saccade amplitude and saccade rate measures suggests important context-dependent influences in changing scanning patterns. Thus, Experiment 2 sought to directly test these influences, and to replicate our null developmental effect for fixation duration and saccade amplitude.

## Experiment 2

Experiment 1 assessed growth trajectories for saccade rate, mean fixation duration, and saccade amplitude and found evidence of moderate to strong individual differences, several of which were stable over time. In addition, these measures were context specific, with patterns changing in complex ways over the course of development. Although Experiment 1 was well-designed for our primary aim of assessing growth trajectories in saccade and fixation metrics, the design of the task makes it difficult to draw firm conclusions regarding the role of content and complexity. Thus, Experiment 2 sought to test the specific relation of content and complexity on scanning patterns using a cross-sectional design with more precisely controlled stimuli, random trial presentation, and slightly longer trials. This design should allow us to determine if scanning patterns vary with content and complexity, and to determine if previous null age effects for fixation duration and saccade amplitude were influenced by our short trial durations and fixed trial order.

### Methods

#### Participants

Infants were tested cross-sectionally at 5, 7 and 11 months. Infant names were obtained from the Tennessee Department of Health, and all infants were full term (gestational age > = 37 weeks) with no reported birth defects or vision problems. The final sample included 72 infants, and all infants were within ± 11 days of the target age. The age distributions were as follows: 5 months (*n* = 24, *M* = 157.42 days, *SD* = 6.30, 15 male, 9 female), 7 months (*n* = 24, *M =* 215.13 days, *SD* = 5.33, 14 male, 10 female), and 11 months (*n* = 24, *M* = 338.17 days, *SD* = 7.23, 9 male, 15 female). An additional 11 infants were excluded due to fussiness, 6 due to sleepiness, 3 due to equipment failure, 1 due to parental interference, 2 due to experimenter error, and 1 due to missing data from 2 or more conditions. Parent reported race and ethnicity for the final sample indicated that 63 of the infants were White (1 Hispanic, 62 Non-Hispanic), 8 were Multi-Racial, and 1 was Black. All mothers reported having at least a high school degree or equivalent, 15 of the mothers reporting having some college or a 2-year degree, 18 had a 4-year degree, 23 had a master’s or professional degree, and 9 had a doctoral degree. Infants received a small toy or t-shirt.

#### Stimuli and procedure

**Fig 3:**
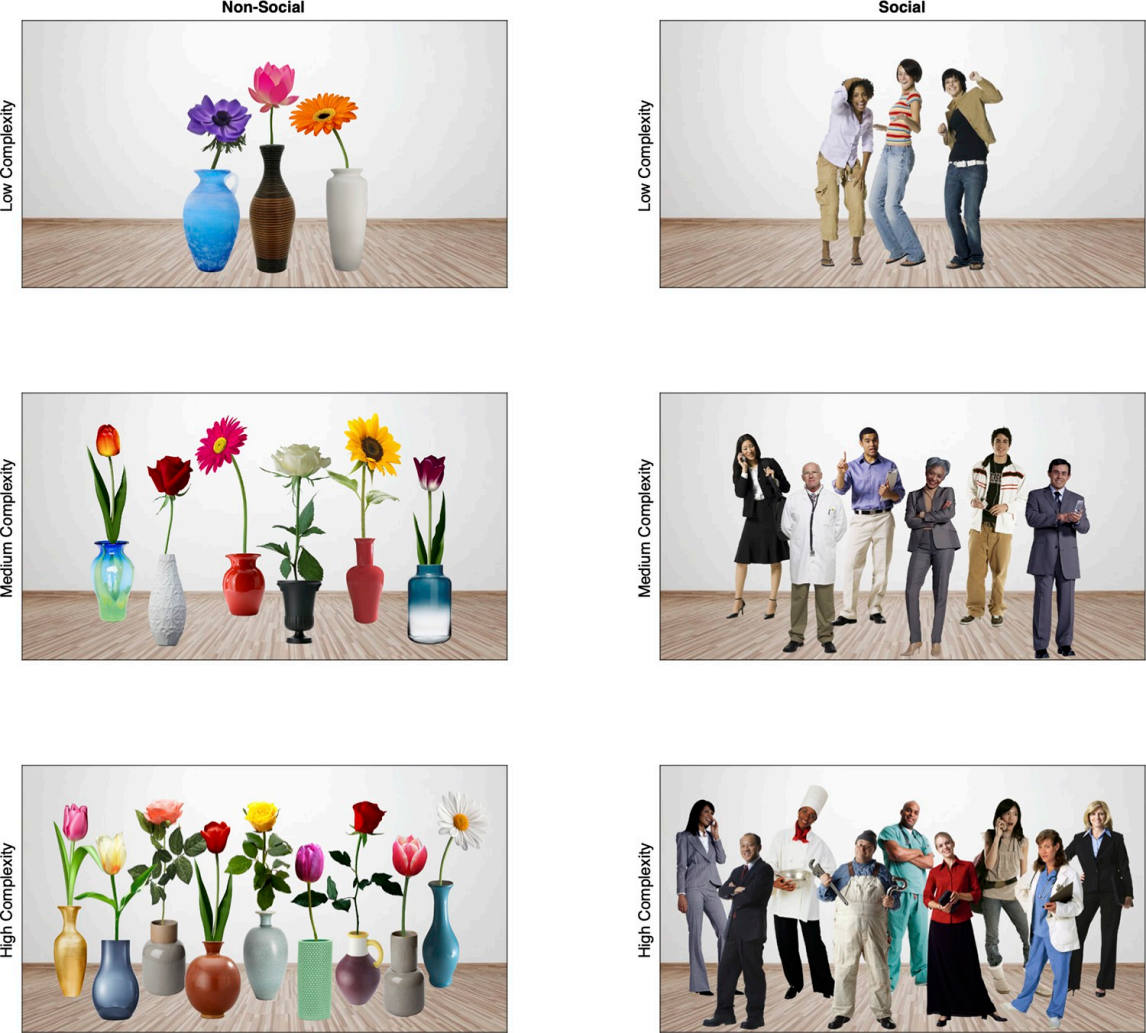
Quasi-naturalistic stimuli used in Experiment 2. As in Experiment 1, scenes varied by content: non-social (left column) or social (right column) and complexity: low (top row), medium (middle row) and high (bottom row). Unlike Experiment 1, trials in Experiment 2 were randomly presented for 10 seconds each and the approximate spatial location of focal points (faces or flowers) was matched across scenes for each level of complexity.

**Fig 4:**
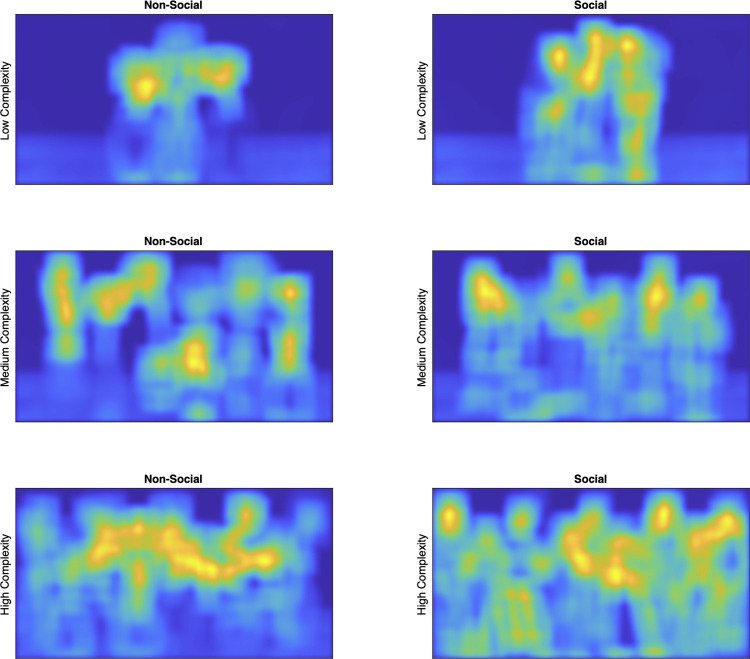
Salience maps of stimuli used in Experiment 2.

The following procedures were approved by the University of Tennessee Institutional Review Board (IRB-17-03545-XP). Written informed consent was obtained from the parent/guardian prior to the testing session. As in Experiment 1, stimuli varied by content (social or non-social) and complexity (low, medium, or high), however we selected flowers in vases as our new non-social objects. Although we explored many non-social stimuli (e.g., clocks, trees, lamps, houses, vehicles), flowers possessed vertical symmetry that was similar to people, were highly salient (compared to trees for example), and had a prominent focal point that could be placed in the same spatial region as the people (Fig 3). The flowers were presented in vases, which roughly equated with the spatial location of the torso and legs of the people, and salience maps revealed reasonably good matches in distribution and salience between the social and non-social stimuli [51; Fig 4]Although it is impossible to equate all the perceptual characteristics that vary between the two distinct classes of stimuli (e.g., luminance and chromatic contrast, internal versus external features, feature density, total contour, familiarity, emotional valence, etc.) we believe the new stimuli roughly controlled for three characteristics most likely to influence our dependent measures: Total eccentricity, focal region, and perceptual salience.

The procedure was nearly identical to Experiment 1. An EyeLink 1000+ remote eye tracker was used to present the stimuli on a 24” 144 Hz color monitor with a viewable surface of 45.5° (w) by 26.76° (h) at a distance of 65 cm. Infants sat on their caregiver’s lap in a dimly lit testing room and point of gaze was sampled using a 16mm lens and 890nm infrared light emitter. Infants were calibrated using a 5-point calibration scheme with dynamic calibration stimuli, and testing did not continue until gaze accuracy was validated to be within 1° for each of the 5 points. As in Experiment 1, infants were shown six trials, three social, three non-social, at varying levels of complexity (low = 3 objects or people, medium = 6 objects or people, high = 9 objects or people, Fig 3) and each trial began with a dynamic audiovisual central fixation stimulus. However, unlike Experiment 1, trials in Experiment 2 lasted 10s and were presented randomly rather than in a fixed order.

#### Eye tracking and data reduction

Continuous gaze was collected monocularly at 500 Hz throughout the session using an EyeLink 1000+ remote eye tracker. Saccades, fixations and blinks were calculated online using EyeLink standard online event parser [52], which incorporates a velocity threshold algorithm to classify saccades with the following settings: Saccade velocity > 40°/s and acceleration > 8000°/s for a minimum duration of 8ms. Samples that did not exceed these thresholds were classified as either fixations (pupil data present) or blinks (no pupil present for at least 3 consecutive samples). Nearby fixations were left unmerged. [53]. Means, standard deviations, and sample sizes for all measures are presented in Table 1.

### Results

#### Linear mixed effects models

The primary aims of Experiment 2 were to replicate major findings from Experiment 1 using a cross-sectional sample, longer trial durations, better stimulus control, and trial randomization. To assess these changes, we once again used LME models [49]. As in Experiment 1, we created three candidate models for each measure, and selected the simplest model that captured the greatest amount of variability. Our first model served as our baseline model (m0) and included a fixed effect of age and a random effect of subject (i.e., random subject-level intercept). Our next model added additional fixed effects for content and complexity, and our final model (m2) added interaction effects for age, content, and complexity (Table 4). Once again, age and complexity were coded as continuous variables and content was categorical (deviation coded so coefficients may be interpreted as main effects).

**Table 4:** Experiment 2 estimates and standard error for all model effects. Significant effects for best-fitting model noted in bold.

	Model Comparisons								
	Saccade Rate (s)			Mean Fixation Duration (ms)			Saccade Amplitude (deg)		
Constant	2.12***	**1.94** ***	1.92***	398.52***	410.28***	**463.10** ***	9.24***	**8.33** ***	7.37***
	(0.12)	**(0.14)**	(0.20)	(29.50)	(34.08)	**(53.31)**	(0.48)	**(0.55)**	(0.87)
Age	-0.13*	**-0.13** ^*^	-0.12	15.96	15.96	-10.45	-0.97***	**-0.97** ***	-0.49
	(0.06)	**(0.06)**	(0.09)	(13.66)	(13.66)	(24.68)	(0.22)	**(0.22)**	(0.40)
Content		**0.29** ***	-0.01		-96.50***	56.15		**1.10** ***	0.68
		**(0.05)**	(0.17)		(13.93)	(49.40)		**(0.22)**	(0.81)
Complexity		**0.09** ^**^	0.10		-5.88	-32.29		**0.46** ***	0.94**
		**(0.03)**	(0.08)		(8.53)	(22.20)		**(0.14)**	(0.36)
Age*Content			0.05			**-35.93** ^*^			-0.03
			(0.06)			**(16.78)**			(0.27)
Age*Complexity			-0.01			13.21			-0.24
			(0.04)			(10.28)			(0.17)
Content*Complexity			0.10			**-40.40** ^*^			0.24
			(0.06)			**(16.78)**			(0.27)
Model	m0	**m1**	m2	m0	m1	**m2**	m0	**m1**	m2
ChiSq Test	--	***p* < .001**	*p* = .301	--	*p* < .001	***p* = .007**	--	***p* < .001**	*p* = .422
Best Fit	no	**yes**	no	no	no	**yes**	no	**yes**	no
Observations	432	**432**	432	432	432	**432**	432	**432**	432
Log Likelihood	-382.09	**-360.61**	-358.79	-2,818.82	-2,796.08	**-2,790.16**	-1,030.26	**-1,013.55**	-1,012.15
Akaike Inf. Crit.	772.19	**733.23**	735.57	5,645.64	5,604.16	**5,598.33**	2,068.51	**2,039.11**	2,042.30
Bayesian Inf. Crit.	788.46	**757.64**	772.19	5,661.91	5,628.57	**5,634.94**	2,084.79	**2,063.52**	2,078.91

Note:

*p<0.05

**p<0.01

***p<0.001

#### Saccade rate

The majority of our model fit metrics converged on model 2 (Table 4). Chi-square analysis further confirmed that model 1 provided a significantly better fit than baseline (*p* < .001), but model 2 did not improve on model fits (*p* = .301). As can be seen in Table 4, estimates from our best-fitting model (m2) once again revealed a significant age main effect, with saccade rates increasing as complexity increased (Fig 2 panel D, panel A). Results also revealed significant content and complexity effects, with higher saccade rates for non-social stimuli, and higher complexity stimuli. These results largely replicate our Experiment 1 findings, though we no longer see an interaction between content and complexity. This may be due to our improved stimulus design.

#### Fixation duration

The majority of our model fit metrics converged on model 2 (Table 4), and our chi-square analysis confirmed this (*p* = .007). Estimates derived from our best-fitting model (m2) reveal a significant main effect of content, with significantly longer mean fixation durations when viewing social stimuli (Fig 2, panel E). In addition, estimates revealed a significant age by content interaction, with 7- and 11-month-old infants showing particularly long fixation durations for social scenes. This is contrary to Experiment 1 and suggests mean fixation durations may actually *increase* with age, albeit in a context-dependent way. Finally, estimates revealed a significant content by complexity interaction, with longest fixation durations for medium and high social scenes (Fig 2, panel E).

#### Saccade amplitude

Results of our model fitting revealed the majority of model fit metrics converged on model 1 (Table 4), and chi-square analysis further confirmed better fits for model 1 (*p* < .001), but not model 2 (*p* = .422). An examination of model estimates for our best-fitting model (m1) revealed a significant age main effect, with younger infants making longer saccades than older infants (Fig 2, panel F). This is distinct from our Experiment 1 results, where we found no age effect. Estimates also revealed both content and complexity main effects, with longer saccades for non-social scenes, and for higher complexity scenes, which is consistent with Experiment 1 findings.

***Infant saccades*: *Costly or compensatory*?** Experiment 2 results reveal that 5-month-olds make more numerous and larger saccades than older infants, but do not have longer mean fixation durations. These differences may be due to relatively unselective and inefficient scene scanning [39]. Given visual systems are essentially blind while engaged in a saccade [54], it is possible that increased saccade rates and amplitudes cause younger infants to spend more time spent with eyes “in flight” decreasing encoding time requiring compensatory increases in overall looking. This could help explain why measures based on holistic attention tend to report longer looking for younger infants, as look durations obtained through standard video coding include *both* fixations and saccades.

To test this, we collapsed scores across condition, and conducted a linear regression examining the relation between age and total fixation duration (summed fixations across each trial). If 5-month-olds compensate for lost encoding time by increasing overall looking, their total looking scores should be higher than older infants. Somewhat surprisingly, results were not significant, *F*(2,69) = 1.080, *p* = .345, *R*^*2*^_*adj*_ = .002, suggesting young infants are not compensating by increasing the amount of time they spend looking overall. An alternative possibility is that younger infants compensate for lost time by producing *faster* saccades. This is plausible, as even young infants show an adult-like saccade “main sequence”, the lawful relation between saccade amplitude and saccade velocity [2, 55–57]. To test this, we again collapsed across condition and conducted a linear regression examining age and peak saccade velocity. The overall model was significant, *F*(2,69) = 6.946, *p* = .002, *R*^*2*^_*adj*_ = .144, with 5-month-olds making significantly faster saccades than 7-month-olds (*p* = .019) and 11-month-olds (*p* < .001). This suggests that although young infants are producing larger, more frequent saccades than older infants, the cost of these increased saccades is partially mitigated by higher saccade velocities. Though others have noted that infants tend to produce faster saccades than adults [2] we are not aware of any other work demonstrating that 5-month-olds produce significantly larger and faster saccades than older infants, a marker of scanning inefficiency [39].

**Fig 5:**
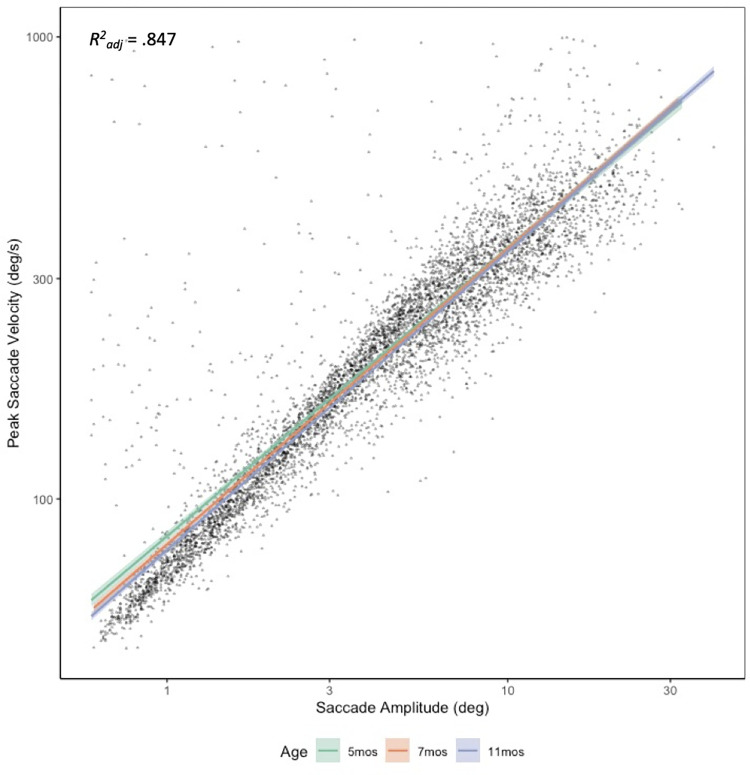
Main sequence and linear regression lines by age. Dots indicate individual observations with peak saccade velocities < 1000 degrees/second and amplitudes >.5 degrees. Shading indicates 95% confidence interval.

We next plotted the infant main sequence to determine if the significant age effect was due to higher proportions of large saccades for our 5-month-old infants, or if 5-month-old infants produce faster saccades even when controlling for saccade size. Peak saccade velocity and saccade amplitude scores were log transformed and filtered to remove blink saccades, saccades away from the monitor, and peak saccade velocities that were less than 1000 degrees/second and amplitudes that were greater than .5 degrees. This helped to reduce erroneous observations due to high frequency eye tracker noise, blinks, and looks away from the monitor. As can be seen in Fig 5, the main sequence was highly consistent across ages, and a regression analysis with a fixed effect of age confirmed a significant overall linear trend, *F*(3,5741) = 10,570, *p* < .001, *R*^*2*^_*adj*_ = .847. Model estimates further revealed that 5-month-old infants (*M* = 103.652 deg/s, *SE* = 2.179) were significantly faster than both 7-month-old (*M* = 95.450 deg/s, *SE* = 2.582), *p* = .029) and 11-month-old infants (*M* = 92.940 deg/s, *SE* = 2.557, *p* < .001, Fig 5). Thus, young infants both produce larger saccades, and have significantly faster saccades than older infants, even when controlling for saccade amplitude. Though others have noted age-related differences in main sequence as a function of attentional engagement [57], our results additionally suggest age-related differences in peak-saccade velocity.

***“Seeing” or “looking”*.** Previous work with adults reveals at least two efficient scanning modes. A passive mode that relies on automatic visual processes and prioritizes *local processing* (i.e., “seeing”), with longer fixation durations and larger subsequent saccades [37], and an active mode which relies on executive processes, and is marked by short fixation durations and short saccades, presumably to increase likelihood of target detection (i.e., “looking”). Although the active strategy seems less likely in infants due to underdeveloped executive attention, it is possible that the high degree of interest and/or perceptual expertise infants have for social content might elicit a more active search strategy, whereas the lower familiarity and semantic salience of the non-social scenes might elicit a more passive strategy. Either way, evidence for one of these strategies would suggest efficient and selective scanning.

**Fig 6:**
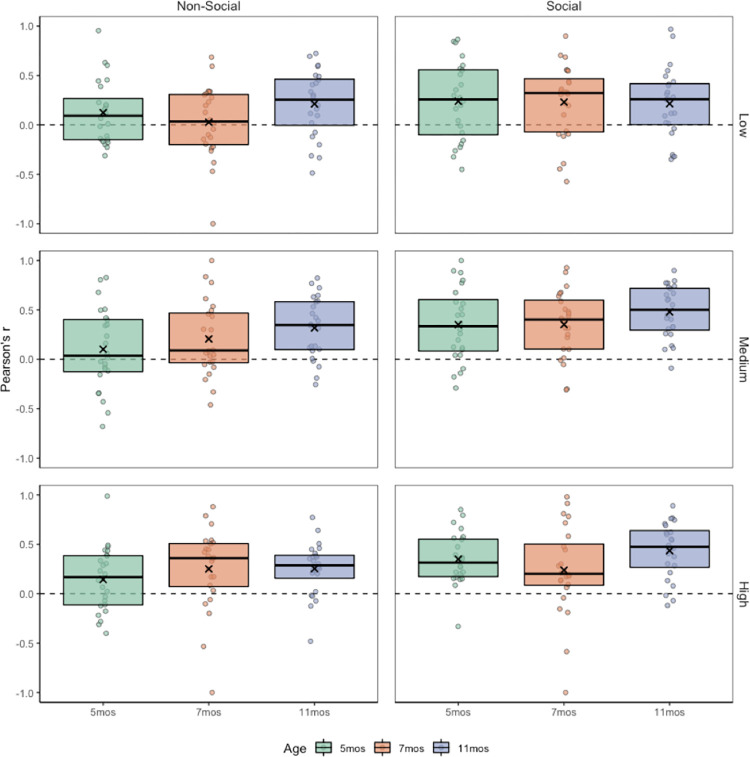
Correlation between fixations and subsequent contiguous saccade amplitudes. Positive correlations reflect high scanning efficiency. Boxplot edges represent upper and lower quartiles, center line represents the median, and ‘X’ represents the mean. Individual dots represent subject-level Pearson correlation coefficients (r). Average number of observations per r as follows: 5 months = 20.28 (SD = 6.18), 7 months = 17.91 (SD = 7.30) and 11 months = 17.69 (SD = 5.08). Asterisks denote significant difference from zero as follows: *p < .05, **p < .01, ***p < .001.

To test this, we conducted a correlational analysis to examine the relation between each individual fixation duration and the amplitude of the saccade *immediately following that fixation*. If infants are engaged in strategic scanning (either active or passive), we would expect to find a consistent, *positive* correlation between individual fixation durations and the immediately subsequent saccade amplitude (i.e., long fixations followed by long saccades and/or short fixations followed by short saccades; *scanning efficiency*). Subject-level Pearson correlation coefficients were conducted comparing the length of each fixation to the length of the immediately subsequent saccade (mean *df* at 5 months = 18.28, 7 months = 17.91, and 11 months = 15.69). These subject-level coefficients were then averaged for each condition to determine if mean coefficients were significantly different from zero using one-sample t-tests. If the majority of individuals are consistent, efficient scanners (i.e., positive correlations), then the mean group correlation should be significantly greater than zero. If, however, the majority of group members are inconsistent or inefficient scanners (i.e., no correlation), we would expect group means to approach zero. As can be seen in Fig 6, 11-month-olds showed high scanning efficiency across all conditions, as evidenced by high subject-level correlation coefficients. In contrast, 7-month-old infants showed low scanning efficiency when viewing low complexity non-social scenes, and 5-month-old infants showed low scanning efficiency when viewing both low and medium non-social scenes. An examination of the means in Fig 6 suggests that across all infants, scanning efficiency was higher for social scenes, and for high complexity scenes. This was confirmed with a 2x3x3 ANOVA, with content (non-social, social) and complexity (low, medium, high) as within subjects variables, and age (5mo, 7mo, 11mo) as a between subjects variable. Results revealed several significant effects, including significant main effects of age, *F*(2,69) = 4.331, *p* = .017, *h*^*2*^ = .112, content, *F*(1,69) = 16.791, *p* < .001, *h*^*2*^ = .196, and complexity, *F*(2,138) = 5.719, *p* = .004, *h*^*2*^ = .077. There were no significant interaction effects, all *p* > .05.

### Discussion

Results from Experiment 2 are numerous and consistent. First, results demonstrate strong content and complexity effects, that cannot easily be attributed to low-level stimulus confounds. These include higher saccade rates and saccade amplitudes, and lower fixation durations for non-social scenes relative to social scenes, and for higher complexity scenes relative to lower complexity scenes. These findings demonstrate that scanning patterns change depending on the content being viewed (social versus non-social) and the complexity of the scene. In addition, though all infants showed evidence of content-dependent scanning pattern changes, saccade rate, amplitude and velocity all *decreased* with development, which is consistent with previous work suggesting skilled scanners produce smaller more frequent saccades [39]. However, mean fixation duration *increased* with development replicating our finding from Experiment 1. Interestingly, this developmental increase is apparent only when viewing social stimuli, suggesting age-related increases in attentional selectivity. Furthermore, this novel finding suggests that our trial durations are sufficiently long to capture developmental effects.

Although 5-month-old infants produced larger more frequent saccades, results from an analysis examining the amount of total looking for each trial (total fixation duration) did not vary by age, suggesting young infants did not compensate for time lost to saccades by increasing their total looking. Rather, it appears that 5-month-old infants produced significant *faster* saccades than both 7- and 11-month-old infants, and these differences were apparent even when controlling for saccade amplitude. Finally, results from our subject-level correlation analysis suggest that all infants are demonstrating some degree of efficient scanning, though to a much larger degree for older infants. Across all infants, this systematicity was most pronounced for social scenes, and for higher complexity scenes suggesting in addition to changing *endogenous* factors (i.e., learning, experience) *exogenous* features such as scene content and complexity can influence scanning strategy.

## General discussion

Infant visual scanning was observed as infants passively viewed scenes that varied in content (social or non-social) and complexity (low, medium, or high). Although our task included a social/non-social manipulation, our intention was not to assess unique patterns of looking to social objects *per se*, this has been well-characterized elsewhere. Rather, our goals were to determine which saccade metrics were most consistent and robust, and if individual differences in scanning systematicity varied by age, scene content, and complexity. Over two experiments, we found evidence for stable individual differences and pronounced developmental differences in several key scanning dynamics, as well as age and context-dependent changes in scanning efficiency. Main results are summarized below.

### Individual differences in saccade dynamics are relatively stable

In Experiment 1, infants were tested longitudinally a 5, 8, and 11 months, and results revealed moderately stable individual differences for several saccade metrics, including saccade rate (5 to 11 months), mean fixation duration (8 to 11 months), and saccade amplitude (5 to 8 months, and 8 to 11 months; Table 3). Results from LME analysis are consistent with these findings and suggest that saccade metrics may be a particularly stable way of tracking individual differences in online cognitive processing (Table 2).

### Younger infants move their eyes more than older infants

Across both Experiments 1 and 2, we found that 5-month-old infants produced more frequent saccades that were larger and faster than older infants, suggesting that 5-month-old infant eye movements are neither slow, nor or hypometric (Figs 2 and 5, panels A and C). Again, this is somewhat contrary to the characterization of young infants as sluggish orienteers in the context of visual competition, though it fits well with findings noting that relatively unskilled scanners are likely to produce larger saccades [39]. What might be driving these effects? It is possible that higher saccade rates at 5 months are the result of an interaction between well-developed reflexive eye movement systems and relatively under-developed saccade inhibition and/or sustained fixation mechanisms [58]. Though automatic saccades can be inhibited either intentionally [59] or through visual competition [6, 60], both of these require maturation of pathways connecting frontal cortical areas to saccade generation areas such as the brainstem and superior colliculus [3].

Although younger infants made larger, more frequent saccades, our results suggest they do not compensate for lost encoding time by increasing overall looking. Rather, our results suggest that infants may compensate by producing *faster* saccades. Five-month-old infants produced significantly faster saccades than both 7- and 11-month-old infants (Fig 5, panel D), and a main sequence analysis suggested these differences were not due to a disproportionate number of large saccades for our youngest infants (Fig 5). Though others have noted the relative speed of infant saccades [2] we are unaware of any other work demonstrating a developmental velocity effect within such a small age range.

Although we found no evidence of developmental decreases in either our mean fixation or summed fixation duration scores, there may be multiple reasons for this. First, our age range may simply have been too narrow. This seems unlikely, as previous research has found developmental differences in the same age range [17], and results from Experiment 2 revealed a significant developmental *increase* in mean fixation duration (Fig 2, panel E). Second, it is possible that our quasi-naturalistic stimuli were either too complex or trials were too brief to reveal individual differences. This too seems unlikely, as others have reported similar null effects using a naturalistic design with brief trial intervals [44]. Third, it is possible that increased eye tracker noise might have produced artificially high saccade rates for our youngest infants [e.g., 47]. However, we found qualitatively similar saccade rates across two commercial eye trackers (Tobii TX300 for Experiment 1, Eyelink 1000+ for Experiment 2) bolstering our confidence that data quality and saccade/fixation parsers are not driving our results. We also found moderate to strong correlations between saccade rate at 5 months and 11 months, and between saccade rate at 5 months and saccade amplitude at 8 months and 11 months suggesting 5-month-old measures were reasonably robust. Finally, despite making more frequent saccades, we see substantially lower variability for 5-month-olds relative to 7-month-olds (Fig 6).

It is possible that individual fixations, the smallest unit of information processing, are qualitatively different than bouts of holistic attention classically measured in familiarization and habituation studies [33]. This seems likely, as each bout of holistic attention typically includes both fixations and saccades (as well as short looks away), and we show here substantial evidence that the frequency and duration of saccades change markedly with development. As others have noted, holistic attention may also reflect vigilance [27, 61], or *oriented focus* that is a crucial component of developing cognitive systems. This possibility will need to be addressed in future research. Despite no evidence of developmental decreases in mean fixation duration, measures based on saccades (frequency, amplitudes, and velocity) show much promise, and may be especially useful when paired with fixation durations to produce a scanning efficiency scores, as summarized below.

### Scanning efficiency varies by stimulus content: Same effect, different mechanism?

Previous work with adults suggests at least two efficient search modes, with active searchers producing frequent, short fixations followed by relatively short saccades (i.e., “looking”), and passive searchers producing relatively infrequent, long fixations and larger subsequent saccades (i.e., “seeing”). Although our subjects are not engaged in an explicit search task, evidence of either of these strategies would suggest some degree scanning efficiency.

Results from our subject-level (Fig 6) correlational analysis comparing fixation duration to subsequent saccade amplitudes suggests the degree of scanning efficiency varies by age, content and complexity. In general, 11-month-olds were the most efficient scanners, followed by 7-month-olds, and finally 5-month-olds. All infants showed higher scanning efficiency for social scenes than non-social scenes, and all infants showed higher scanning efficiency for high complexity scenes than for low complexity scenes. What might be driving these context effects? There are several possible factors that may have contributed to our content effect, including differences in semantic salience for social versus non-social scenes, increased interest/arousal, general familiarity, perceptual expertise, and reflexive orienting to social stimuli [62]. Though our results cannot distinguish from these possibilities, others have found that relative interest and attentional engagement can influence the relation between saccade size and speed (i.e., “*main sequence*”) [57]. In addition to scene content, scene complexity also influenced scanning efficiency, with higher efficiency for higher complexity scenes. It is unlikely that perceptual expertise, familiarity, or semantic salience differences can account for this effect. It may be the case that low complexity scenes simply do not elicit strategic scanning because they are less interesting, or perhaps because the stimuli are relatively sparce. Alternatively, it may be the case that increased visual competition for higher complexity scenes inhibits reflexive orienting [60] requiring additional top-down control of eye movements [6], although this cannot explain our content effect. Might two distinct mechanisms be driving scanning efficiency? Or are both subserved by a similar mechanism such as arousal or overall salience? Future work is currently underway to address these possibilities.

It is important to note that our scanning efficiency correlation analyses were based on the assessment of individual fixation/saccade pairs. That is, we compared each individual fixation to the immediately subsequent saccade. This is important, as *mean* fixation duration and *mean* saccade amplitudes reveal a different pattern, with older infants showing relatively long fixation durations and short saccade amplitudes, and younger infants trending towards relatively short fixation durations and long saccade amplitudes (Fig 2, panels E and F). Although overall means highlight important developmental changes, scanning *efficiency* measures are unique, and necessarily require examination at the level of each individual fixation and the subsequent contiguous saccade.

### Are infants “seeing” or “looking”?

Results from Experiment 2 demonstrate developmental differences in scanning efficiency, with a positive correlation between saccade amplitude and fixation duration, particularly when viewing higher complexity scenes, and social scenes (Fig 6). However, it is difficult to determine if this relation was driven by infants producing *longer* fixations followed by *longer* saccades (“seeing”), or *shorter* fixations followed by *shorter* saccades (“looking”). Given this was not an explicit search task, the most conservative approach might be to assume that infants are engaging in a passive “seeing” strategy driven primarily by automatic visual processes. Certainly, the relatively long mean fixation durations (Fig 2, panel E), coupled with relatively high scanning efficiency (Fig 6) support this possibility, at least while viewing the social scenes. However, we cannot rule out the possibility that increases in visual competition for the higher complexity conditions might invoke more top-down “executive” orienting processes. Future work will address this question.

In conclusion, our results demonstrate that 5-month-old scanning patterns are less efficient, resulting in more frequent saccades that are higher in both amplitude and velocity than 7- and 11-month-old infants. Results also demonstrate that individual differences in saccade rate and saccade amplitude show moderate stability from 5 to 11 months, and both of these metrics vary with scene content and complexity. Scanning efficiency is also influenced by scene content and complexity, with infants showing the most efficient systematic scanning when viewing social and higher complexity scenes, and with 11-month-olds showing the most consistent efficient scanning. Taken together, results provide strong evidence of both developmental and context-dependent changes in scanning efficiency, and suggest scanning metrics hold much promise as markers of cognitive processing and individual differences.
